# Specific oral immunotherapy versus allergen avoidance for food allergy in children: systematic review and meta-analysis (update)

**DOI:** 10.1186/2045-7022-3-S3-P2

**Published:** 2013-07-25

**Authors:** C Iliescu, C Lamotte, C Preda

**Affiliations:** 1 Pediatrics, Allergology Centre, Catholic Hospitals of Lille, France; 2 Internal Medecine, Universitary Hospital of Lille, France; 3 Biostatistics, Polytech University 1, Lille, France

## Background

Specific oral immunotherapy was reported as an alternative to dietary allergen avoidance in children with food allergy. Our aim was to assess the effectiveness and safety of specific oral tolerance induction (SOTI) compared to allergen avoidance in children and adolescents with Immunoglobulin E-mediated food allergy.

## Methods

### Design

Systematic review and meta-analysis.

### Data sources

Medline (1992 to October 2012), reference list of retrieved articles.

## Study selection

Randomized controlled trials (RCT) of SOTI versus allergen avoidance with the following criteria: clinical history of Immunoglobulin E mediated food allergy diagnosed using double blind placebo-control food challenge; three phases SOTI protocol (induction, build-up, maintenance); children aged 0-18 years; final status (tolerance/allergy) and safety data reported in both groups. Two authors independently extracted data.

## Results

Five RCTs met our inclusion criteria. Four studies showed a statistically significant reduction in endpoint allergy after SOTI versus control. All pooled analysis were based on random-effects models. A significant heterogeneity between the studies was observed (Q = 31.79, p < 0.001; I^2^ = 87.4%, 95% confidence interval (CI), 73-94.1%). The relative risk (RR) of allergy was lower after SOTI (RR = 0.36; 95% CI, 0.18-0.75). The meta-regression showed no relation between SOTI efficacy (log(RR)) and the duration of the protocol or the age of patients. Compared with control, the RR of adverse events during SOTI ranges from to 0.18 to 2.33 and no significant correlation with RR of allergy is detected.

## Conclusion

Our meta-analysis (240 children), considers two additional RCTs (240 children) with respect to the Fisher’s one (2010). It supports the evidence that SOTI decreases the risk of food allergy in children despite the heterogeneity of the different studies.. Furthermore, the effectiveness of SOTI to induce tolerance in children with food allergy is not associated with a higher tendency of adverse events during the protocol compared to allergen avoidance.

## Disclosure of interest

None declared.

